# Comparison of the Gut Microbiota of Patients Who Improve with Antibiotic Combination Therapy for Ulcerative Colitis and Those Who Do Not: Investigation by Fecal Metagenomic Analyses

**DOI:** 10.3390/nu16203500

**Published:** 2024-10-16

**Authors:** Toshifumi Ohkusa, Kimitoshi Kato, Tsuyoshi Sekizuka, Toshiro Sugiyama, Nobuhiro Sato, Makoto Kuroda

**Affiliations:** 1 Department of Microbiota Research, Juntendo University Graduate School of Medicine, Tokyo 113-0033, Japan; nsato@juntendo.ac.jp; 2 Division of Gastroenterology and Hepatology, Department of Internal Medicine, The Jikei University School of Medicine, Kashiwa Hospital, Kashiwa, Chiba 277-8567, Japan; 3 Division of Research Planning and Development, Nihon University School of Medicine, Tokyo 173-8610, Japan; vyk01273@nifty.com; 4 Pathogen Genomics Center, National Institute of Infectious Diseases, Tokyo 162-8640, Japan; sekizuka@nih.go.jp (T.S.); makokuro@nih.go.jp (M.K.); 5 Advanced Gastrointestinal Cancer Molecular Targeted Therapy and Prevention Research Division, Hokkaido University Hospital, Sapporo 060-8648, Japan; toshisugi5397@ac.cyberhome.ne.jp

**Keywords:** ulcerative colitis, antibiotic combination therapy, metagenomic analysis

## Abstract

**Background/Objectives**: The cause of ulcerative colitis (UC) may be related to commensal bacteria in genetically susceptible patients. We previously demonstrated that triple antibiotic combination therapy induces remission in patients with active UC in randomized controlled trials (RCTs). Now, we investigate changes in the gut microbiota of patients who responded to the antibiotic combination therapy. **Methods**: Thirty-one patients with UC given ATM/AFM (amoxicillin, metronidazole, and tetracycline or fosfomycin) therapy for two weeks were enrolled in this study. The clinical conditions of these UC patients were evaluated by the partial Mayo score. The gut microbiota was compared via the metagenomic shot gun analysis of fecal samples. **Results**: Of the 31 patients, 16 and 8 experienced complete and partial remission, respectively, over three months in response to ATM/AFM therapy, whereas ATM/AFM showed no efficacy in 7 patients. The dysbiosis before treatment in the active stage could be associated with increased populations of *Bacteroides*, *Parabacteroides*, *Rickenella*, *Clostridium*, *Flavonifractor*, *Pelagibacter*, *Bordetella*, *Massilia*, and *Piscrickettsia* species. Metagenomic analysis revealed dramatic changes in the gut microbiota at an early stage, that is, just two weeks after starting ATM/AFM therapy. After treatment in the responder group, the populations of bifidobacterium and lactobacilli species were significantly increased, while the population of bacteroides decreased. **Conclusions**: These results suggest that metagenomic analysis demonstrated a marked change in the gut microbiota after antibiotic combination treatment. In the triple antibiotic combination therapy, remission was associated with an increase in bifidobacterium and lactobacilli species.

## 1. Introduction

There is increasing evidence suggesting that the intestinal flora plays an important role in the pathogenesis of ulcerative colitis (UC) [1]. In patients susceptible to intestinal infections, cell-mediated immune responses to dysbiosis of the intestinal bacterial component are excessively enhanced, resulting in intestinal inflammation and pathogenesis [2]. The profile of the gut microbiota differs substantially between patients with active UC and healthy controls [1]. Accordingly, altering the gut microbiota of patients with UC through appropriate antibiotic therapy against microbial pathogens may lead to improvement and remission of active UC.

In our earlier reports, *Fusobacterium varium* (*F. varium*) was found to be present in the colonic mucosa of UC patients, with a high frequency of 84% [3]. Furthermore, butyrate, a product of the culture supernatant of *F. varium*, was shown to cause UC-like lesions in mice [4]. Based on the results of these studies, a multicenter RCT was undertaken to determine the efficacy of a regimen of combination antimicrobial agents (amoxicillin, tetracycline, and metronidazole (ATM) for 2 weeks) against microbial pathogens of UC, including *F. varium*, in the remission and maintenance of active UC [5]. Compared with a placebo, ATM therapy produced more effective improvements in endoscopic and Mayo scores, remission, and steroid withdrawal in patients with active UC. Furthermore, we showed that a multicenter, long-term follow-up study suggests that two-week antibiotic combination therapy (ATM) is effective and safe in patients with active UC refractory or dependent on steroids. The changes in the intestinal microbiota associated with antibiotic-ATM therapy were evaluated by terminal restriction fragment length polymorphism (T-RFLP) of mucosal-associated bacterial components. ATM therapy induced long-term changes in the intestinal microbiota of UC patients [6], which may be associated, at least in part, with the clinical effects of the therapy. Recently, we modified ATM therapy to AFM therapy (amoxicillin, fosfomycin, and metronidazole). AFM therapy is effective for treating UC, as is ATM therapy [7].

Recently, fecal transplant therapy has been used as a treatment targeting intestinal bacteria for ulcerative colitis, but there are reports of both effectiveness and ineffectiveness, and no conclusion has been reached [1,8]. However, in studies in which not only fecal transplant therapy but also an antibiotic combination therapy similar to ATM/AFM therapy was administered before FMT for ulcerative colitis, a response rate of over 50% has been reported [9].

To date, a comprehensive evaluation of microbiological dynamics via the metagenome has not been performed between patients who are in remission, who have been in remission, and who have been in remission due to ineffective antibiotic combination therapy. Recently, it has been reported that intestinal bacteria are involved in the therapeutic response to immunotherapy using anti-PD1 antibodies against cancers [10]. Therefore, we characterized the bacterial taxonomic and functional changes associated with antibiotic combination therapy (ATM and AFM) via shotgun sequencing and the metagenomic analysis of fecal samples. Fecal samples were obtained prior to, during, and 3 months after antibiotic combination treatment.

## 2. Materials and Methods

### 2.1. Patient Study Subjects

A multicenter, open-label study was conducted at 3 hospitals (Jikei University Kashiwa Hospital, Nihon University Hospital, and Hokkaido University Hospital) in Japan. The institutional review board or ethics committee at each facility approved the protocol. All the subjects provided written informed consent. All eligible patients had an established diagnosis of UC. Study subjects were selected from among patients with chronic relapsing or continuous UC who had received 5-ASA therapy, steroid therapy, immunomodulator therapy, or anti-TNF therapy and who regularly visited outpatient clinics or were hospitalized. Patients were excluded if they had toxic megacolon or penicillin allergy, were pregnant, had severe liver or kidney disease, or had a psychiatric illness. Patients were also excluded if they had taken antibiotics within 4 weeks before study entry or were infected with *Clostridium difficile* or other fecal pathogens at the time of entry.

### 2.2. Study Design

The patients received a combination antibiotic regimen comprising oral amoxicillin (1500 mg/day), tetracycline (1500 mg/day) or fosfomycin (3000 mg/day), and metronidazole (750 mg/day) (ATM/AFM) for two weeks. Treatment for UC was provided on routine clinical grounds using sulfasalazine, 5-aminosalicylic acid, prednisolone, azathioprine (AZA), and an anti-TNF antibody (infliximab). Standard doses of these medications were given at least one month prior to enrollment. The doses and administration of these drugs, except for prednisolone, were then maintained throughout the study period, and when symptoms improved, the prednisolone dose was tapered by 5 mg per week from week 8 onwards until it reached 20 mg per day. Thereafter, the dose was reduced by 2.5 mg per week until it was discontinued. No probiotics or prebiotics were administered after the antibiotic combination therapy.

At each assessment, patients completed a symptom questionnaire and underwent clinical examinations to determine partial Mayo scores [11]. According to the partial Mayo score, 2–4 were mild, 5–6 were moderate, and 7 or more were severe. Partial Mayo scores provide an assessment of disease activity based on a combination of symptoms and signs, with scores ranging from 0–9. Clinical response was defined by a reduction in the partial Mayo Clinic score of at least 3 points and a decrease of at least 30%, with a decrease of at least 1 point on the rectal bleeding subscale or an absolute rectal bleeding score of 0 or 1 from the baseline score for UC patients. Clinical remission was defined as a partial Mayo Clinic score ≤ 2 and a combined stool frequency and rectal bleeding subscore ≤ 1 [12]. Those that deviated from the score defined above were regarded as nonresponsive.

Clinical relapse was defined as the return of visible blood in the stool for 2 consecutive days and/or the recurrence of frequent diarrhea (6 or more bowel movements per day), nocturnal diarrhea, or abdominal cramps. If a patient relapsed or developed severe or fulminant UC symptoms, the study was discontinued and appropriate treatment was administered. Patients visited the clinic weekly or monthly and were evaluated by clinical examination. Patients were classified as steroid refractory if their condition had not improved after at least 2 weeks of intravenous or oral administration of more than 30 mg/day prednisolone, and patients were classified as steroid dependent if they had experienced a relapse while tapering prednisolone to at least 10 mg/day and were unable to discontinue steroids without relapse.

### 2.3. Sample Collection

Fecal samples were obtained from each patient prior to, after, and 3 months after ATM/AFM therapy. The feces from each patient were frozen at −80 °C until use.

### 2.4. DNA Isolation from Fecal Samples

Patient feces (approximately 100 mg) were suspended in 10 mL of Tris-EDTA buffer (pH 7.5), 50 μL of 100 mg/mL lysozyme type VI purified from chicken egg white (MPBIO, Derby, UK), and 50 μL of 1 mg/mL purified achromopeptidase (Wako, Osaka, Japan) were added, followed by incubation at 37 °C for 1 h with mixing. Next, 0.12 g of sodium dodecyl sulfate (final conc. 1%) was added, and the suspension was mixed until it became clear. Finally, 100 μL of 20 mg/mL proteinase K (Wako) was added, followed by incubation at 55 °C for 1 h with mixing. Crude nucleic acid purification was performed with phenol–chloroform extraction, and the RNA was subjected to ethanol precipitation and dissolved in 1.6 mL of ASL buffer from a QIAamp DNA Stool Mini Kit (QIAGEN, Tokyo, Japan). To ensure that the removal of inhibitors from the molecular biology experiments occurred in the feces, the extracted DNA sample was purified with a QIAamp DNA Stool Mini Kit (QIAGEN), according to the manufacturer’s instructions.

### 2.5. Whole-DNA Sequencing and Metagenomic Analyses

DNA libraries were prepared using a NexteraXT DNA Sample Prep Kit (Illumina-compatible, EPICENTRE Biotechnologies, Madison, WI, USA), and all of the sequencing runs were performed with a MiSeq (Illumina, San Diego, CA, USA), according to the manufacturer’s instructions. All of the obtained DNA sequencing reads were aligned to a reference human genomic sequence using BWA-MEM read-mapping software [13], with quality trimming to remove low-quality bases and adapter sequences. The remaining sequence reads were subjected to a megaBLAST search against four public databases (nt, refseq genomic, Representative_Genomes, and other_genomic). For the taxonomic assignment of reads, these homology search results were analyzed using MEGAN version 6.7.0 [14], with a minimum support of 1 hit and a minimum score of 150. Short reads assigned to bacterial taxa were extracted, followed by normalization of the metagenomic data to the number of one million reads. The beta diversity indices were analyzed with “betadiver” in the R vegan package [15], and principal coordinate analysis (PCoA) was performed using the “plot” function. A statistical analysis of the bacterial community and a classification of the metadata were performed, and permutational multivariate analysis of variance (PERMANOVA) was performed using 10,000 permutations and the Bray–Curtis dissimilarities method with “adonis” in the R vegan package. A metagenomic biomarker discovery approach, LEfSe, was used to determine the differences in the abundances of bacterial species in the fecal samples of UC patients before treatment and ≥3 months after antibiotic administration. For linear discriminant analysis effect size (LEfSe), Kruskal–Wallis and pairwise Wilcoxon tests were performed, followed by linear discriminant analysis (LDA) to assess the effect size of each differentially abundant taxon [16]. In this study, a *p* value < 0.05 was considered to indicate statistical significance for both methods. Bacteria with markedly increased numbers were defined as those with an LDA score (log10) of more than 2.

### 2.6. Data Deposition

Short-read sequences, including excluded human sequences, were deposited with the Japanese DNA Data Bank.

### 2.7. Statistical Analysis

We used the Mann–Whitney U test to determine the significance of differences in age, disease duration, Mayo score, and Mayo endoscopic score between the response and nonresponse groups. The chi-square test and Fisher’s exact test were used to compare sex, extent of disease, clinical severity of disease, steroid use, and medication use between the two groups. All calculations were performed using STAT VIEW software, version J 5.1 (SAS Institute, Inc., Cary, NC, USA). A *p* value < 0.05 was considered to indicate statistical significance.

## 3. Results

### 3.1. Baseline Characteristics

We enrolled 31 patients with active UC (Table 1; median age 45 years, range 22–66 years, male/female ratio 17/14) whose disease was severe (*n* = 2), moderate (*n* = 25), or mild (*n* = 4) according to the criteria of the partial Mayo score. The left side and extensive colitis type accounted for the majority of the cases. Four patients with steroid-refractory UC and fourteen patients with steroid-dependent UC were included.

We observed a clinical response in terms of the partial Mayo score at 2 weeks and 3 months after the administration of the antibacterial agent, and 24 patients comprised the responder group (Table 1). However, ATM/AFM showed no efficacy in seven patients in the nonresponder group. There were no significant differences in age, sex, disease duration, partial Mayo score, Mayo score, Mayo endoscopic score, extent or severity of disease, steroid use, or medication between the responder and nonresponder groups.

### 3.2. Metagenomic Analyses

The results of the shotgun metagenomic analyses of fecal samples were compared between the two groups who received ATM/AFM therapy before treatment, two weeks after the end of medication, and three months after treatment.

**Figure 1:**
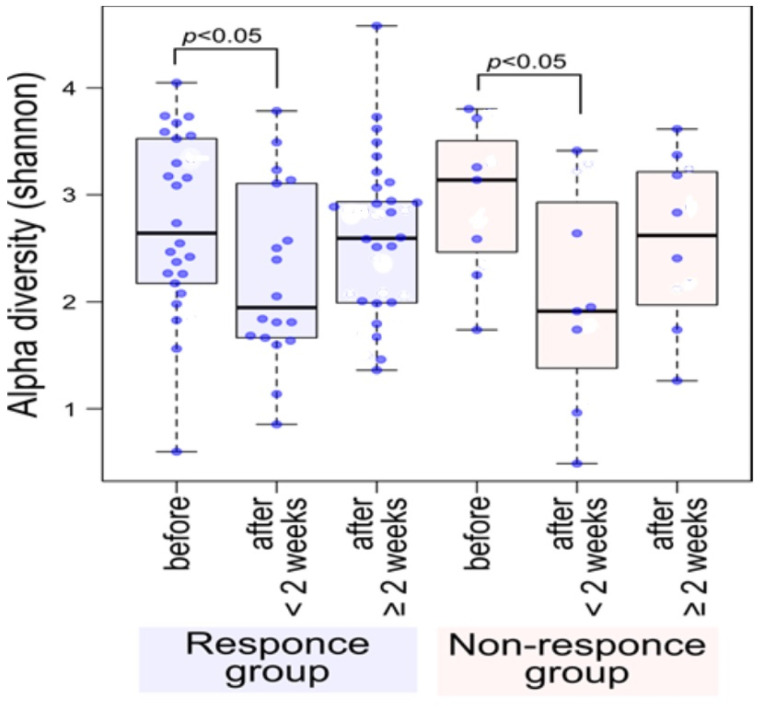
Shannon alpha diversity showed a significant decrease after the end of medication in patients in the response and nonresponse groups compared to the baseline before treatment (PERMANOVA *p* < 0.05). The Shannon index was recovered at 3 months after antibiotic administration for both responders and nonresponders. Although there was significant variation in the bacterial flora due to the antibacterial agents, the bacterial flora diversity tended to generally recover after treatment. Blue dots indicate data for each subject.

**Figure 2:**
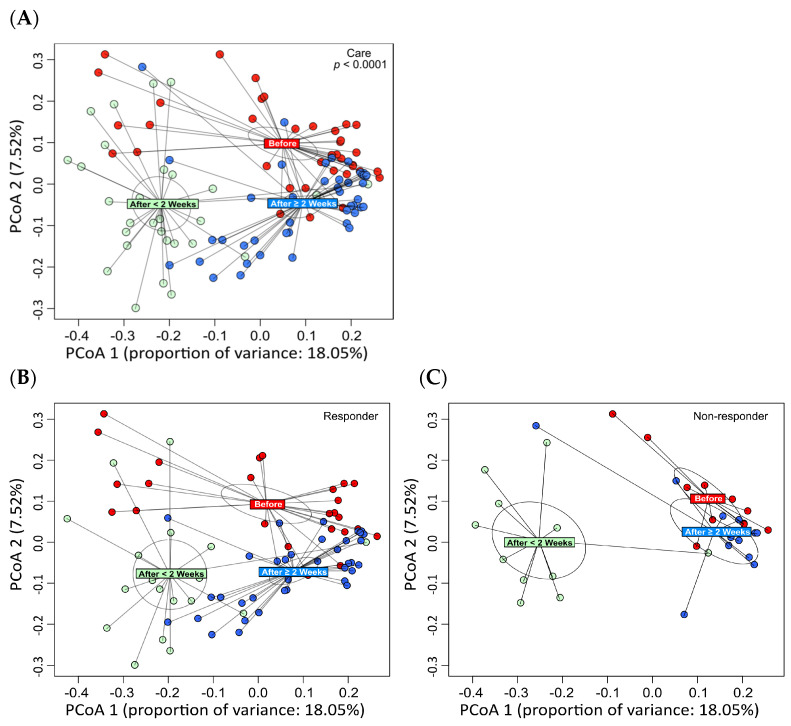
Principal coordinate analysis (PCoA) of the bacterial community taxonomic structures at the species level using beta diversity distances. The colored rectangles indicate the centroid of each classification. PCoA revealed significant changes in the gut microbiota at the end of ATM/AFM therapy and at 3 months after antibiotic administration compared to before antibiotic administration (**A**). The composition of the bacterial flora changed before and after treatment for 3 months due to the administration of the antibacterial agent in the responder and nonresponder groups (**B**,**C**). The difference in the abundances of bacteria in the responder group suggests that different bacterial flora may have formed 3 months after treatment. However, there were no significant differences in beta diversity before or 3 months after treatment in the nonresponder group.

Dramatic changes in the gut microbiota were observed at an early therapeutic stage, i.e., just two weeks after starting ATM/AFM therapy (Figure 1 and Figure 2), suggesting that antibiotic therapy strongly influenced the bacterial community structures. Fecal alpha diversity, which evaluates species richness and evenness, was also analyzed; at baseline, the responder group could not be distinguished from the nonresponder group (Figure 1). Shannon diversity indices were significantly lower after the end of medication in patients in the response and nonresponse groups than at baseline (PERMANOVA *p* < 0.05). The Shannon index was recovered at 3 months after antibiotic administration for both responders and nonresponders. Although there was significant variation in the bacterial flora due to the antibacterial agents, the bacterial flora diversity tended to generally increase after treatment.

Principal coordinate analysis (PCoA) was performed on the bacterial community taxonomic structures at the species level using beta diversity distances (Figure 2). The colored rectangles indicate the centroid of each classification. PCoA revealed significant changes in the gut microbiota at the end of ATM/AFM therapy and at 3 months after antibiotic administration compared to before antibiotic administration (Figure 2A). The composition of the bacterial flora changed before and after treatment for 3 months due to the administration of the antibacterial agent in the responder and nonresponder groups (Figure 2B; Figure 2C). In the responder group, different bacterial flora may have formed 3 months after treatment. However, there were no significant differences in beta diversity before or 3 months after treatment in the nonresponder group.

### 3.3. Linear Discriminant Analysis Effect Size (LEfSe) at the Species Level

**Figure 3:**
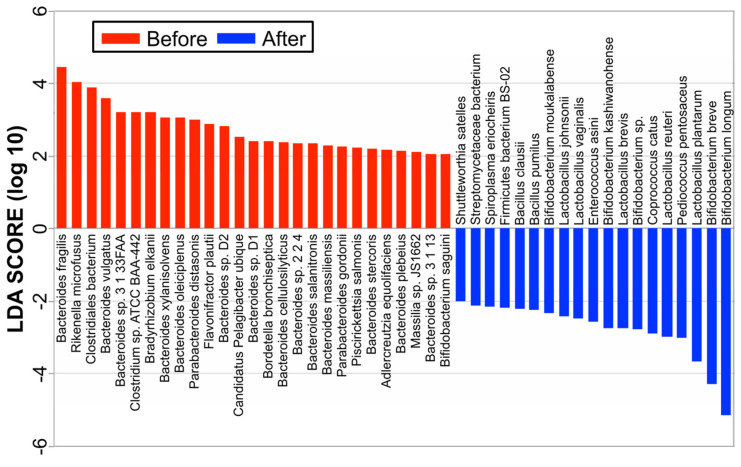
LEfSe at the species level. A comparison of the metagenomic data between the responder group before treatment and 3 months after antibiotic administration suggests that dysbiosis before treatment in the active stage was possibly associated with increased populations of *Bacteroides, Parabacteroides*, *Rickenella*, *Clostridium*, *Flavonifractor*, *Pelagibacter*, *Bordetella*, *Massilia*, and *Piscrickettsia* species. In responders after treatment, the populations of *Bifidobacterium* and *Lactobacillus* species were significantly increased.

Linear discriminant analysis (LDA) combined with effect size measurements (LEfSe) revealed a list of features that enabled discrimination between before treatment and 3 months after the treatment phase in the fecal samples. A comparison of the metagenomic data between the responder group (Figure 3) before treatment and 3 months after antibiotic administration suggests that the dysbiosis before treatment in the active stage was possibly associated with increased populations of *Bacteroides*, *Parabacteroides*, *Rickenella*, *Clostridium*, *Flavonifractor*, *Pelagibacter*, *Bordetella*, *Massilia*, and *Piscrickettsia* species. In responders after treatment, populations of *Bifidobacterium* and *Lactobacillus* species (*B. longum*, *B. breve*, *L. plantarum*, *L. reuteri*, *L. brevis*, *B. kashiwanohense*, *L. vaginalis*, *L. johnsonii*, and *B. moukalabense*) were significantly increased, while the population of Bacteroides was decreased. On the other hand, in the nonresponder group, before the antibiotics treatment, populations of *Bacteroides*, *Parabacteroides*, *Rickenella*, *Clostridium*, *Flavonifractor*, *Pelagibacter*, *Bordetella*, *Massilia*, and *Piscrickettsia* species were also increasing, but there was no significant change in the abundances of gut microbiota components, such as *Bacteroides*, *Parabacteroides*, *Bifidobacterium*, and *Lactobacillus* species after the treatment.

## 4. Discussion

The intestinal microbiota varies considerably with respect to dysbiosis between active UC patients and healthy controls [1,2]. Therefore, appropriate antibiotic therapy against microbial pathogens could alter the intestinal microbiota in patients with UC, resulting in the improvement and remission of active UC. Our previous studies revealed significant clinical and endoscopic improvement in active UC after treatment with the combination of antibiotics, ATM and AFM [5,7]. Recently, similar ATM therapies have been reported to be effective for refractory pediatric UC patients [17,18,19,20]. Antibiotics were given orally in these studies. On the other hand, the administration of multidrug antibacterial agents by intravenous injection instead of oral administration was reported to be ineffective in an RCT for adult ulcerative colitis [21]. The greater effectiveness of oral administration than intravenous administration suggests that antibacterial agents altered the intestinal flora and improved UC incidence. After five days of treatment, in a similar ATM therapy for pediatric acute severe ulcerative colitis [18], the mean pediatric ulcerative colitis activity was significantly lower than that in the control group. In the fecal microbiome before treatment, the highly dominant genera were *Escherichia*, *Haemophilus,* and *Enterobacter*, all of which belong to the class *Gammaproteobacteria* and rarely dominate the healthy gut microbiome. However, during antibiotic treatment, an increase in taxa belonging to *Gammaproteobacteria* (mainly *Escherichia* and *Haemophilus*) was observed, coupled with a decrease in multiple taxa belonging to the *Clostridiales* and *Bacterodiales* orders (to which most known butyrate producers belong). At first remission, none of the control patients had *Proteobacteria* blooms, whereas several patients who received antibiotics continued to display *Proteobacteria* dominance, which was also more common in the antibiotic group at baseline. Therefore, the dominance of *proteobacteria* in the gut microbiota is not necessarily a bad thing. In our previous studies of ATM therapy, we altered the profile of terminal restriction fragment length polymorphism (T-RFLP) in mucosa-associated bacterial components and reduced mucosal bacteria and the abundance of *Fusobacterium varium* [22]. According to a real-time polymerase chain reaction (PCR) study, only *F. varium* was significantly reduced after treatment [6]. As previous studies have indicated, there is a reduced diversity of bacteria and an increased abundance of *Fusobacterium* in the gut of UC patients [23,24,25]. Regarding *Fusobacterium*, a study of FMT in UC patients showed that those who did not achieve remission had an increased level of *Fusobacterium* in their gut microbiota [26]. In this study, the fecal microbiota contained very few *Fusobacteria*. The low detection rate of *Fusobacteria* may be because the number of bacteria in feces is low, many dominant bacteria, such as *Bacteroides*, are detected, and *Fusobacteria*, which have a low prevalence, are ignored. In fact, *Fusobacteria* are often detected in ulcerative colitis patients through the analysis of not fecal but mucosal microbiota [4,6,25]. Additionally, there was a decrease in the abundances of *Bacteroidetes* and *Lactobacillus* species, although the effects on the Firmicutes group remain controversial [27,28].

Antibiotic combination therapy can induce and maintain significant alterations in the mucosal microbiota in patients with UC at least 3 months after treatment [6]. These results suggest that antibiotic combination therapy changed and reduced the mucosal and fecal microbiota abundances after treatment. However, the above research involved 16S metagenomic analysis rather than shotgun metagenomic analysis of the whole genome. This study used shotgun metagenomic analyses, and there were no metagenomic data on changes in the microbiota after antibiotic combination therapy in active UC patients.

This study revealed that 17 and 7 UC patients with partial Mayo scores experienced complete and partial remission, respectively, over three months in response to ATM/AFM therapy, respectively. The microbial taxonomic changes before and after treatment with antibiotic combination therapy according to metagenomic analysis were identified and associated with therapeutic responders and nonresponders. In both groups, dramatic changes in the gut microbiota were observed at an early therapeutic stage, that is, just two weeks after starting ATM/AFM therapy, suggesting that antibiotic therapy strongly influenced the bacterial community structures. The Shannon index alpha diversity of the microbiomes recovered after ATM therapy at 3 months after antibiotic administration in both responders and nonresponders. However, in the responder group, different bacterial flora may have formed 3 months after treatment, but there were no significant differences in beta diversity before or 3 months after treatment in the nonresponder group. According to the analysis at the bacterial genus level by LEfSe analysis, comparison of the metagenomic data suggests that dysbiosis before treatment in the active stage could be associated with increased populations of *Bacteroides*, *Parabacteroides, Rickenella*, and *Clostridium* at the phylum level; however, in the remission phase, three months after the end of ATM/AFM therapy, the ratio of *Bifidobacterium* to *Lactobacillus* bacteria increased markedly. It has been reported that an alteration in the intestinal microbiota may cause a breakdown of barrier function, such as an abnormal immune response to normal flora, and result in UC [1,2]. The intestinal epithelium serves as a barrier between the luminal environment and the mucosal immune system and guards against harmful molecules and microorganisms. Beneficial bacteria and probiotics promote mucosal health by strengthening barrier integrity, increasing local defenses (mucin and IgA production), and inhibiting proinflammatory immune responses and apoptosis to promote mucosal homeostasis. In contrast, pathogenic bacteria and pathobionts suppress the expression and localization of tight junction proteins, cause dysregulation of apoptosis/proliferation, and increase proinflammatory signaling that directly damages the intestinal mucosa [29].

The mucosal barrier function chiefly consists of tight junctions, a multistrain probiotic (*Lactobacillus plantarum*, *Streptococcus thermophilus*, and *Bifidobacterium breve*). *Lactobacillus paracasei*, *Lactobacillus delbrueckii* subsp. *bulgaricus*, *Lactobacillus acidophilus*, *Bifidobacterium longum*, and *Bifidobacterium infantis*) upregulated the expression of junctional proteins following 6 weeks of probiotic therapy [30]. Additionally, *Bifidobacterium longum* has been reported to improve intestinal barrier function and suppress inflammation [28]. It is suspected that three months after the end of ATM/AFM therapy in the responder group, increases in bifidobacterium and lactobacillus bacteria recover the mucosal barrier and may cause an improvement in UC. Lactobacillus and bifidobacteria are well-known probiotics, and it was interesting that the results were consistent with those of the responder group. In the future, it may be possible to further strengthen supplementation of the Bifidobacteria and Lactobacilli combination (*B. longum*, *B. breve*, *L. plantarum*, *L. reuteri*, *L. brevis*, *B. kashiwanohense*, *L. vaginalis*, *L. johnsonii*, *B. moukalabense*) after antibiotic combination therapy as a new treatment method. Ishikawa et al. compared AFM therapy alone and received AFM therapy followed by FMT, and they reported that the response and remission rates were higher in the group that also received FMT [31]. This suggests that administering the above-mentioned probiotics after antibiotic therapy may be effective.

One limitation of our study is the small number of cases; however, it could be small because shotgun metagenomic analysis is very expensive. In addition, this study analyzed the intestinal bacteria involved in the effect of antibiotic therapy by comparing responders and nonresponders, so controls did not seem to be necessary.

## 5. Conclusions

Metagenomic analysis demonstrated a marked change in the gut microbiota after antibiotic combination treatment. In the triple antibiotic combination therapy, remission was associated with an increase in bifidobacterium and lactobacilli species after the treatment. It may be possible to further strengthen supplementation of the bifidobacteria and lactobacilli combination after antibiotic combination therapy as a new treatment method.

## Figures and Tables

**Table 1:** Baseline characteristics of patients.

	Total	Responder	Nonresponder
Median age, year (range)	45 (22–66)	45 (22–66)	47 (23–53)
Male/female, number of patients	17/14	12/12	5/2
Median disease duration, years (range)	8 (0–30)	10 (0–30)	4 (1–9)
Partial Mayo score, median (range)	5 (2–9)	5 (2–9)	7 (5–9)
Mayo score, median (range, *n* = 28)	8 (4–12)	7 (3–12)	9 (6–12)
Mayo endoscopic score, median (range, *n* = 28)	2 (1–3)	2 (1–3)	2 (1–3)
Extent of disease, number of patients			
Extensive colitis	20 (65%)	17	3
Left-side colitis	10 (32%)	6	4
Proctitis	1 (3%)	1	0
Clinical severity of disease, number of patients			
Severe	2 (7%)	1	1
Moderate	25 (81%)	19	6
Mild	4 (13%)	3	0
Steroid use, number of patients			
Steroid dependent	17 (55%)	10	7
Steroid resistant	4 (13%)	3	1
None	10 (32%)	8	2
Medication, number of patients			
Sulfasalazine	5 (16%)	5	0
5-ASA	24 (77%)	17	7
Corticosteroid	5 (16%)	4	1
thiopurine	4 (13%)	2	2
anti-TNF	2 (6%)	1	1

## Data Availability

Short-read sequences, including excluded human sequences, have been deposited in the Japanese DNA Data Bank.
